# Expanding the Genotypic and Phenotypic Spectrum of *OFD1*-Related Conditions: Three More Cases

**DOI:** 10.3390/genes15121633

**Published:** 2024-12-20

**Authors:** Tatiana Kyian, Artem Borovikov, Inga Anisimova, Oksana Ryzhkova, Maria Bulakh, Elizabeth Bragina, Maria Avakyan, Anna Demchenko, Victoria Zabnenkova, Victor Kovalev, Artem Bukhonin, Elena Kondratyeva, Sergey Kutsev

**Affiliations:** 1 Research Centre for Medical Genetics, 1 Moskvorechye St., 115522 Moscow, Russiademchenkoann@yandex.ru (A.D.); a.v.bukhonin@gmail.com (A.B.);; 2 Belozersky Institute of Physico-Chemical Biology, Lomonosov Moscow State University, Leninskiye Gory, 1, Build. 40, 119991 Moscow, Russia; 3 Saint Petersburg State Budgetary Healthcare Institution “Children’s City Hospital of St. Olga”, 2 Zemledelcheskaya St., 194146 Saint-Petersburg, Russia

**Keywords:** primary ciliary dyskinesia, oro-facio-digital syndrome type 1, Simpson–Golabi–Behmel syndrome type 2, ciliopathy, sequencing, electron microscopy, high-speed video microscopy, *OFD1*

## Abstract

Introduction: Pathogenic variants in the *OFD1* gene are linked to a spectrum of syndromes that exhibit partial clinical overlap. Hemizygous loss-of-function variants are considered lethal in males, while heterozygous loss-of-function variants generally result in oro-facial-digital syndrome type 1. A reported phenotype, Simpson–Golabi–Behmel syndrome type 2, was published once but remains controversial, with many specialists questioning its validity and arguing about its continued listing in the OMIM database. Methods: To investigate the genetic and phenotypic characteristics of the patients, we performed clinical exome sequencing, family-based genetic analysis, X-inactivation studies, electron microscopy, and detailed clinical assessments. Results: Three patients from unrelated families carrying loss-of-function variants in the *OFD1* gene were identified, emphasizing the diverse phenotypic spectrum of *OFD1*-associated disorders. The first patient, a female with a heterozygous frameshift variant p.(Gln398LeufsTer2), was diagnosed with oro-facial-digital syndrome type 1. The second patient, a male with a heterozygous nonsense variant p.(Gln892Ter), presented with features resembling Simpson–Golabi–Behmel syndrome type 2, as previously reported under this diagnosis. The third patient, a male with another heterozygous nonsense variant p.(Glu879Ter), exhibited isolated primary ciliary dyskinesia without any syndromic features. Conclusions: This study contributes to the growing body of evidence on the expanding phenotypic spectrum of *OFD1*-associated disorders. It underscores the need for further investigation into the molecular mechanisms underlying the diverse presentations and the necessity of re-evaluating diagnostic classifications for conditions such as SGBS2 in the context of variants in the *OFD1* gene.

## 1. Introduction

The *OFD1* gene consists of 23 exons and 22 introns, located on the short arm of the X chromosome X p22.2, and it encodes a protein of 1012 amino acids [1]. *OFD1* is one of the genes that escapes X-chromosome inactivation, leading to biallelic expression [2]. It encodes a centrosomal protein that localizes in basal bodies, essential for the formation of primary cilia [3]. Hemizygous loss-of-function (LoF) variants are considered embryolethal, while heterozygous variants result in oro-facial-digital syndrome type 1 (OFD type 1) characterized by facial anomalies, internal organ malformations, intellectual developmental delays, and respiratory manifestations [1,4,5].

Truncated variants in the *OFD1* gene are associated with two X-linked recessive phenotypes: Joubert syndrome 10 (JS10), primary ciliary dyskinesia (PCD), and retinitis pigmentosa 23 (RP23) [6,7,8] JS10 is characterized by craniofacial abnormalities, oral abnormalities, retinal dysfunction, cystic disease, skeletal defects, brain malformations, the molar tooth sign, cognitive impairment, and situs inversus [7]. RP23 is a type of inherited retinal dystrophy characterized by the progressive degeneration of the retina. The condition typically leads to a gradual loss of vision, starting with night blindness and peripheral vision loss, and eventually progressing to central vision impairment [9].

PCD involves a motility defect of cilia in the respiratory epithelium, as well as in other structures such as the flagella of spermatozoa, the villi of the fallopian tubes, and the ependyma of the brain ventricles [10]. While most cases of PCD are inherited in an autosomal recessive pattern, X-linked forms also exist. Rarely, heterozygous variants in the FOXJ1 gene or hemizygous variants in the *PIH1D3* or *OFD1* genes have been reported in males [11,12]. It is believed that different truncated variants impact the function of the OFD1 protein differently, as demonstrated in the case of JS10, where the interaction between OFD1 and LCA5-encoded Lebercilin plays a key role in the phenotype [7].

Another syndrome, Simpson–Golabi–Behmel syndrome type 2 (SGBS2), is an extremely rare X-linked recessive condition linked to the *OFD1* gene in the OMIM database [13]. However, many authors consider this association erroneous, arguing that it should not be listed in the OMIM database [5,14]. This viewpoint is primarily based on a single observation of one patient with SGBS2 in 2006, without substantial supporting evidence for additional patients exhibiting a similar clinical picture or significant differences from Simpson–Golabi–Behmel syndrome type 1. Only one additional study of a patient cohort has mentioned a second patient in supplementary tables, but it did not provide detailed clinical descriptions [15].

This article presents the clinical and genetic features of three patients with different pathogenic variants in the *OFD1* gene and varying phenotypic manifestations.

## 2. Materials and Methods

This study involved three patients from unrelated families. All patients (or their parents/legal representatives) provided signed informed voluntary consent to participate in the scientific study and for the use and publication of their medical data (results of examination, treatment, and observation).

### 2.1. Transmission Electron Microscopy

Transmission electron microscopy (TEM) was employed for analysis. Brush biopsy samples were fixed in a 2.5% solution of glutaraldehyde in 0.1 M of cacodylate buffer for 24 h. Ultrathin sections were obtained using a Reichert Jung ultramicrotome, Ultracut E (Reichert Jung, Vienna, Austria), with a diamond knife (Ultra 35deg Diamond Knife 1.5 mm, DiATOME, Nidau, Switzerland). The specimens were examined using a transmission electron microscope JEM-1011 (JEOL, Akishima, Japan) and equipped with an Orius SC1000 W camera (Gatan Inc., Pleasanton, CA, USA). Ultrastructural research was supported by the Moscow State University Development Program (PNR 5.13).

### 2.2. Cell Culture

Primary human nasal epithelial cells (hNECs) were obtained from a human nasal biopsy. Brush biopsies were obtained from slightly changed mucous membranes during the period of remission of the disease using a disposable cytology brush with a working length of 1150 mm, a diameter of 5.0 mm, for a 2.0 mm channel BC-202D-5010 (Olympus Medical Systems Corp., Tokyo, Japan). Brush biopsies were placed in DMEM (PanEco, Moscow, Russia) with 50× penicillin–streptomycin (PanEco, Russia) and 1000× fungin (InvivoGen, France), and transported at +4 °C. Freshly isolated hNECs were cultured on a Matrigel-coated culture dish in PneumaCult™-Ex Plus Medium (StemCell Technologies, Vancouver, BC, Canada) with 1 µM of A83-01 (StemCell Technologies, Vancouver, BC, Canada), 1 μM of DMH1 (Sigma Aldrich, St. Louis, MO, USA), 0.2 μM of Hydrocortisone (StemCell Technologies, Vancouver, BC, Canada), and 100× penicillin–streptomycin.

Differentiation of hNECs into ciliary cells was carried out using the air–liquid interface (ALI) culture method. hNECs were seeded on 6.5 mm Transwells with 0.4 μm of pore (StemCell Technologies, Vancouver, BC, Canada) previously coated with Marigel (Corning, Glendale, AZ, USA) in PneumaCult™-Ex Plus Medium with all supplementary. Once the cells reached a monolayer, the medium in upper chamber was completely removed and the medium in the lower chamber was switched to PneumaCult™-ALI Medium (StemCell Technologies, Vancouver, BC, Canada), 4 μg/mL of heparin (StemCell Technologies, Vancouver, BC, Canada), 9.6 μg/mL of hydrocortisone, 1μM of DAPT (StemCell Technologies, Vancouver, BC, Canada), and 100× penicillin–streptomycin. ALI cultivation was carried out for 24 days with medium replacement every 48 h.

### 2.3. DNA Analysis

For molecular genetic studies, blood samples were collected from the affected patients and healthy family members. Genomic DNA was extracted using standard methods. An exome sequencing was performed for all patients. Target enrichment was performed using Illumina TruSeq^®^ ExomeKit (Illumina, San Diego, CA, USA), and custom oligonucleotides (IDT xGen Exome Research Panel v1.0) included coding regions of over 20,000 protein-coding genes. Paired-end sequencing (2 × 150 bp) was carried out on an Illumina NextSeq 500. The sequencing data were processed using Illumina’s Basespace software (Enrichment 3.1.0). Variant filtering was based on their frequency, with variants having a frequency of less than 1% in the Genome Aggregation Database (gnomAD v.2.1.1) and coding region sequence effects such as missense, nonsense, coding indels, and splice sites being considered. The clinical significance of the variants was evaluated using ACMG criteria for variant interpretation [16]. To validate the pathogenic variant in the affected patient and the family members, Sanger sequencing was performed using the Applied Biosystems 3130 xl Genetic Analyzer (HITACHI, Applied Biosystems Group of The Applera Corporation Japan, Waltham, MA, USA). All variants in the *OFD1* gene were validated by Sanger sequencing, and family analysis was available for 2 of 3 families.

### 2.4. X-Inactivation Analysis at the HUMARA Locus

The unequal X-chromosome inactivation pattern was analyzed according to Allen et al. 13 in 1992. For this purpose, the X-linked HUMARA polymorphic repeat ((CAG)n in AR gene exon 1) methylation pattern was detected using methyl-sensitive quantitative fluorescent PCR (QF-PCR) with subsequent fragmentary analysis on the ABI3130xl Genetic Analyzer (Applied Biosystems, Foster City, CA, USA). The inactivated X chromosome-carrying (with or without the mutant allele) cell percentage (XCI ratio) was evaluated according to the formula proposed by V. Bolduc et al. [17,18].

## 3. Results

### 3.1. Clinical Case N°1

A 6-year-old female was referred to our center for evaluation due to phenotypic anomalies and developmental concerns. The patient was born following an uncomplicated second pregnancy, delivered naturally at 39 weeks of gestation with an Apgar score of 8/8. Her birth weight was 3240 g, and her length was 52 cm. Early motor milestones were age-appropriate; however, significant delays in speech development were noted, with verbal communication limited to babbling.

From the neonatal period, the patient exhibited multiple oral anomalies, including oral cavity bands and tongue lobulation. A plastic surgery performed at 1 year of age revealed histological evidence of a hamartoma. Subsequent surgical procedures at ages 2 and 3 addressed additional hamartomas and oral cavity anomalies, including the frenulum of the tongue and upper lip.

The family history was unremarkable for genetic disorders, except for a half-sibling on the maternal side diagnosed with autism spectrum disorder who lacked notable phenotypic abnormalities.

**Figure 1:**
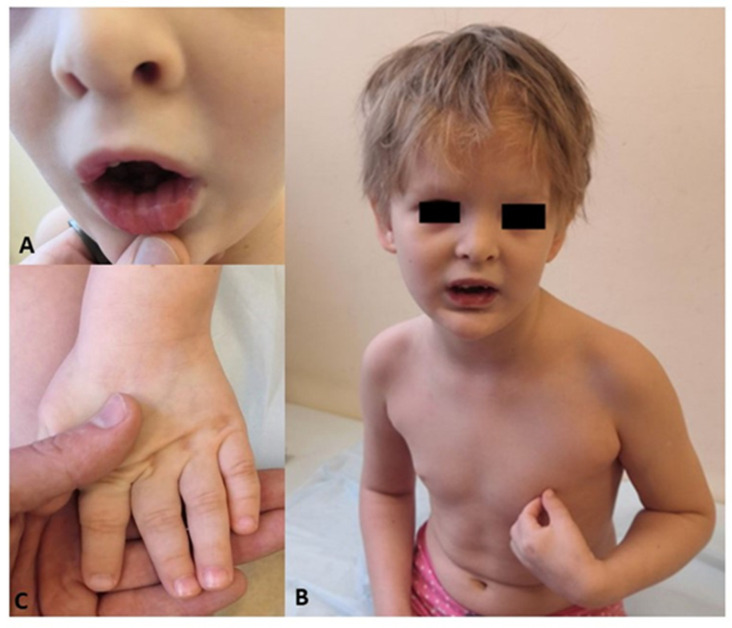
Features of the phenotype of patient #1. (**A**)—Lips with lip pits. (**B**)—Face image shows hypertelorism, thin eyebrows, a wide bridge of the nose, and low-set auricles. (**C**)—Hands imaging shows brachydactyly.

Clinical examination: 6 years old, body weight 24.5 kg (+1.4 SD), height 124 cm (+1.8 SD). Phenotype: dryness in the periorbital area, head circumference 53 cm (+1.5 SD), hypertelorism > 3 SD (distance between the pupils 54.1 mm, between the outer corners of the eyes 83.9 mm, inner 35.1 mm), telecanthus, thin eyebrows, high palate, low-set auricles, wide bridge of the nose, short neck, brachydactyly <-3 SD (length of the hand 10.4 cm, length of the first finger 5 cm, palm 5.4 cm). The patient was friendly, capable of engaging with others, and able to follow instructions, but she remained nonverbal. Neurological examination did not reveal any additional deficits. The features of the phenotype are shown in Figure 1A–C).

An MRI of the brain revealed nodular subependymal heterotopia of gray matter. Video EEG monitoring did not detect any epileptiform activity.

The patient underwent clinical exome sequencing due to her phenotypic features, which were suggestive of oro-facio-digital syndrome. A previously reported heterozygous frameshift variant was identified in exon 12 of the *OFD1* gene, located on chromosome X (chrX:13773332CAATC>C; NM_003611.3:c.1193_1196del; p.(Gln398LeufsTer2)).

A segregation analysis demonstrated that the variant was maternally inherited. Given the healthy status of the mother, we investigated the possibility of imbalanced X-chromosome inactivation (XCI). An analysis of peripheral blood revealed a highly skewed XCI pattern of 98:2. This finding further supports the pathogenicity of the variant and aligns with a diagnosis of OFD type 1, consistent with ACMG criteria (PM2, PVS1, PP5).

### 3.2. Clinical Case N°2

A 16-year-old boy, the first case of this disease in his family, was born from the first pregnancy, which proceeded without complications. Delivery was urgent and assisted with forceps. At birth, his weight was 4200 g and his length was 53 cm. His postnatal condition was extremely severe, with an Apgar score of 1. He presented with apnea, central nervous system (CNS) suppression, myoclonic seizures, and congenital pneumonia, requiring artificial lung ventilation for nine days. He was discharged on day 21.

Early development was marked by a perinatal hypoxic-traumatic CNS injury, accompanied by muscular dystonia and persistent regurgitation. Delayed motor and speech development were evident. By two years of age, he exhibited an unsteady gait with elements of frontal ataxia. At three years old, he was diagnosed with cerebral palsy (ataxic form), bulbar–pseudobulbar syndrome, atonic-asthenic syndrome, and vocal cord paresis.

The patient exhibited a daily productive cough and persistent rhinitis since birth. By the first year of life, he required repeated hospitalizations due to pneumonia. Shortness of breath during physical activity and persistently decreased blood oxygen saturation (SpO_2_) levels of 88–90% were noted. A chest CT scan performed at 5 years old revealed atelectasis of the middle lobe of the right lung, a consolidation zone in the fifth segment of the left lung, and multiple peribronchial thickenings in the basal lung sections. An echocardiogram at 6 years old showed left ventricular dilation.

Since the age of 7, the patient exhibited symptoms such as “drumstick” fingers and “watch glass” nails, consistent with chronic hypoxia. Spirometry at the same age showed a forced vital capacity (FVC) of 37% and forced expiratory volume in one second (FEV1) at 45% of predicted values. At the age of 8, aortography revealed hyperplastic bronchial vessels, and the patient underwent selective embolization of the bronchial arteries on three occasions (at ages 7, 8, and 10) due to episodes of hemoptysis.

At 16 years old, 12 h pulse oximetry revealed episodes of nocturnal desaturation, with SpO_2_ levels below 90e% recorded during 43% of the sleep period. Clinical management improved after introducing continuous oxygen therapy during sleep.

Additional evaluations revealed hypermetropic astigmatism, divergent strabismus, and congenital cataracts diagnosed by an ophthalmologist at 10 years old. The otorhinolaryngological examination confirmed chronic otitis media and bilateral conductive hearing loss (Grade 1 on the right and Grade 2 on the left). A chest CT scan at 16 years old revealed atelectasis of the middle lobe and lingular segments with traction bronchiectasis.

**Figure 2:**
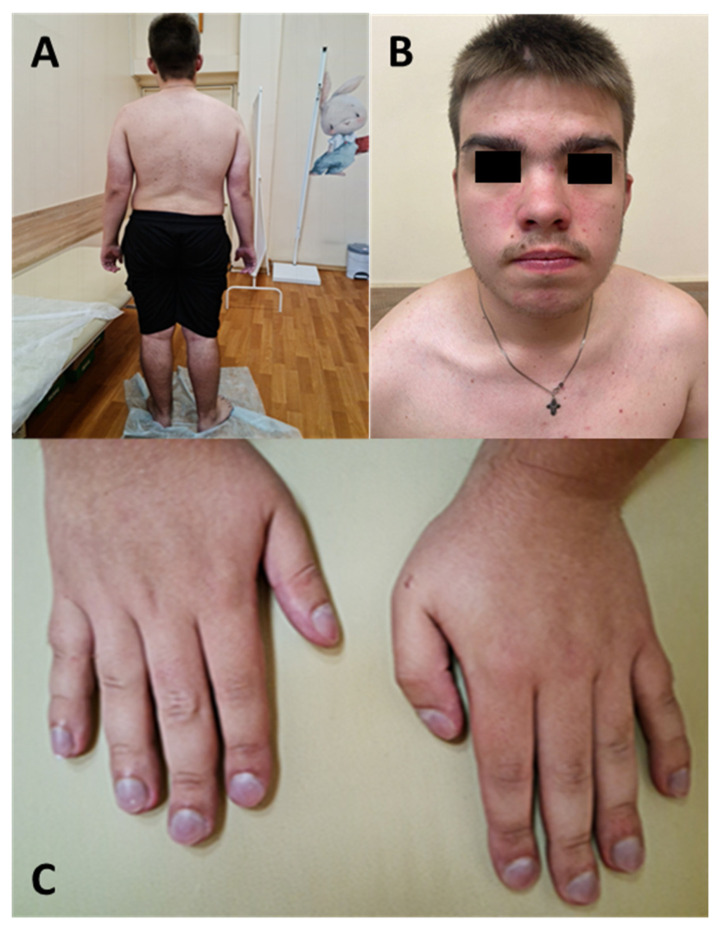
Phenotypic features of patient #2. (**A**)—Body posture imaging demonstrates high body weight and scoliosis. (**B**)—Facial image reveals a high forehead, wide protruding mandible, full cheeks, a wide nose with a broad flat nasal bridge, a large mouth, and low-set ears. (**C**)—Hand imaging shows wide palms and camptodactyly.

Clinical examination at the age of 16 years and 9 months revealed the following: height 177 cm (+1 SD), body weight 95 kg (+2.1 SD), head circumference 61 cm (+4.1 SD), and BMI 30.3 kg/m^2^ (+2.2 SD) (Figure 2). The respiratory rate was 20 breaths per minute, the pulse was 70 beats per minute, and the SpO_2_ saturation was 91–92%. The patient exhibited difficult nasal breathing. Auscultation revealed stiff breathing with crepitating rales across the entire lung surface.

Molecular genetic testing revealed a normal male karyotype (46, XY). Genetic analyses for Prader–Willi and Martin Bell syndromes, as well as MLPA for common deletions associated with Smith–Magenis syndrome, showed no significant findings. Sweat tests for cystic fibrosis were within normal limits, measuring 34 and 32 mmol/L. Subsequent whole exome sequencing identified a previously unreported hemizygous nonsense variant in exon 20 of the *OFD1* gene (chrX:13767201C>T; NM_003611.3:c.2674C>T; p.(Gln892Ter)). The patient’s mother was found to be a heterozygous healthy carrier of this variant. Based on ACMG criteria (PVS1, PM2), the variant was classified as likely pathogenic, and a diagnosis of SGBS2 type 2 was suggested.

Following the WES results, a brush biopsy of the nasal epithelium was performed for light video microscopy, air–liquid interface (ALI) cultures to assess ciliary function, and transmission electron microscopy (TEM). In light microscopy of the native ciliated epithelium and ALI cultures, no visible ciliary movements were recorded. The “program for determination of ciliary epithelium beat frequency in primary ciliary dyskinesia” was used to assess ciliary motion. The ciliary beat frequency was significantly reduced, measured at 3.5 ± 0.8 Hz in the biological preparation and 3.9 ± 0.6 Hz in ALI cultures, compared to 7.3 ± 2.1 Hz and 8.6 ± 2.47 Hz, respectively, in the control group. These findings confirmed ciliary dyskinesia. Transmission electron microscopy revealed the absence of inner dynein arms, a hallmark of PCD. In some cilia, inner dynein arms were detected sporadically in single doublets.

### 3.3. Clinical Case N°3

The patient is a 14-year-old male who presented with persistent wet cough and difficulty breathing. His family history is significant for bronchial asthma, affecting his mother, grandmother, and great-grandmother. He was born following a complicated first pregnancy with toxicosis and a threatened termination, resulting in delivery at 39 weeks gestation. His neonatal period was uneventful. Early in childhood, he was evaluated by a local neurologist due to a speech delay.

At 3.5 years of age, he developed bronchitis and bilateral pneumonia, requiring hospitalization and antibiotic treatment. Following the pneumonia, he experienced recurrent episodes of bronchitis (up to 4–5 times annually), often associated with broncho-obstructive syndrome. Starting at the age of 5, increased sputum production was noted. The patient also had a history of otitis, though hearing loss was not observed.

**Figure 3:**
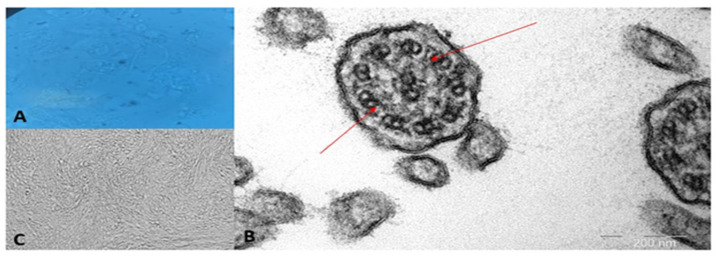
Characterization of ciliary cells. (**A**)—Ciliated cells in the native preparation; (**B**)—Transmission electron microscopy of the cilia of the respiratory epithelium. Inner dynein arms are absent; (**C**)—ALI-culture of ciliated cells. Red arrows indicate space where inner dynein arms should be.

**Figure 4:**
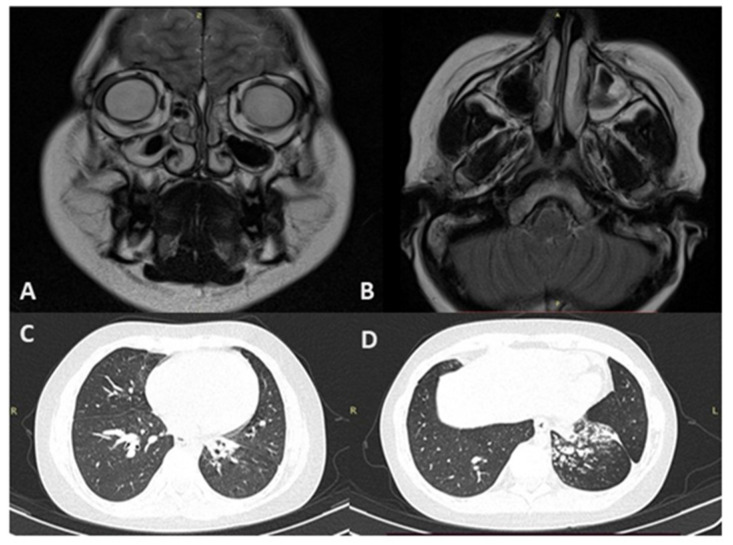
MRI of the paranasal sinuses (**A**,**B**) and CT scans of chest organs (**C**,**D**).

At age 10, there was a significant increase in cough frequency and sputum production, prompting a pulmonology consultation. To rule out cystic fibrosis, molecular genetic testing for 12 common pathogenic variants in the CFTR gene was performed, which did not reveal pathogenic variants in heterozygous or homozygous state. Additionally, a sweat test was conducted, yielding negative results (43 mmol/L). The patient has been under the care of a pulmonologist since age 11, with a diagnosis of chronic bronchitis. A scraping of the mucous membrane from the right and left nasal passages was performed, revealing a marked decrease in motile ciliated cells, along with group and cellular asynchronism, indicative of dyskinesia (Figure 3). A computed tomography (CT) scan of the paranasal sinuses performed at age 12 demonstrated rhinosinusitis involving both maxillary sinuses and the ethmoid labyrinth, with the presence of a fluid pathological component. An MRI of the paranasal sinuses conducted at age 13 revealed parietal thickening of the maxillary sinus mucosa, accompanied by a minimal fluid level in the left sinus, as well as involvement of the ethmoid cells and mammillary cells of the temporal bones. (Figure 4A,B).

At 14 years old, decreasing respiratory function was detected, with FVC at 72% of the predicted value and FEV1 at 74% of the predicted value. Bronchoscopy revealed signs of purulent endobronchitis in the left lower lobe bronchus, as well as bilateral serous–mucous endobronchitis. Indirect signs of bronchiectasis were observed in the lower lobe of the left lung. Total IgE levels were normal, and IgE specific to Aspergillus fumigatus in 2023 was 0.03 (N ≤ 0.35 IU/mL). CT scans of the chest showed signs of low-intensity foci and areas of consolidation in segments S2, S5, and S6 of the right lung, as well as in S6 of the left lung. Bronchiectasis, pneumofibrosis, and changes in the lower lobe of the left lung were noted. The scans also revealed consolidation in S5 of the right lung, signs of bronchiolitis in the lower lobe of the left lung, and pleuroapical, pleuropulmonary, and pleurodiaphragmatic adhesions in both lungs (Figure 4C,D).

At 14 years old, the patient’s examination revealed a respiratory rate of 19 breaths per minute, a heart rate of 88 beats per minute, an SpO2 of 99%, and a blood pressure of 110/70 mmHg. The PICADAR scale score was four, indicating a moderate clinical condition. The patient did not experience shortness of breath but had a persistent wet productive cough. Breathing was slightly labored with clear exhalation, and occasional dry rales were heard without specific localization. Percussion sounds were noted to be boxed, and heart sounds were rhythmic and sonorous with no murmurs detected.

Whole exome sequencing revealed a previously unreported hemizygous nonsense variant in exon 20 of the OFD1 gene—chrX:13785281G>T; NM_003611.3:c.2635G>T; p.(Glu879Ter). According to the ACMG criteria (PVS1, PM2), this variant was classified as likely pathogenic.

**Figure 5:**
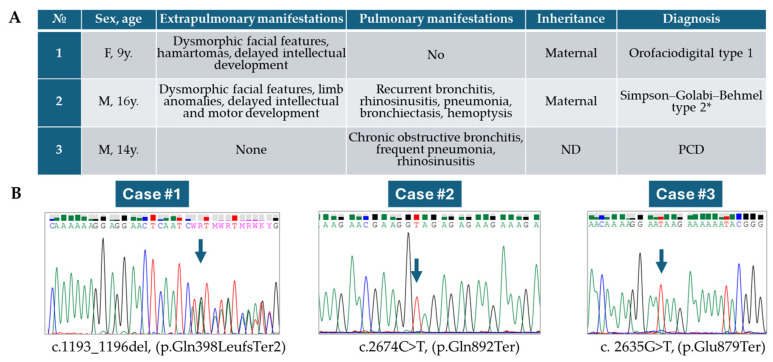
(**A**) Summary of diagnosis and clinical findings from all patients in this study. (**B**) Sanger chromatogram, validation results after NGS for all patients. F—female, M—male, y.—years old at time of last examination, ND—no data. Arrows indicate positions of identified variants. *—The term “Simpson–Golabi–Behmel syndrome type 2” is used here to denote its place within the phenotype spectrum of OFD1-associated disorders, although its clinical appropriateness may require further clarification.

Summary of clinical data of all patients presented in Figure 5.

## 4. Discussion

In our article, we summarized our observations on the clinical and genetic features of three patients with genetic syndromes associated with variants in the *OFD1* gene. The spectrum of *OFD1*-associated syndromes includes both overlapping and isolated conditions, which makes differential diagnosis particularly challenging [5].

In the first case, we describe a female patient with a heterozygous frameshift variant in the *OFD1* gene p.(Gln398LeufsTer2) that was maternally inherited. The presence of this frameshift variant in a healthy mother prompted an investigation of XCI, which revealed a highly skewed XCI pattern of 98:2 in the patient’s peripheral blood, while the mother exhibited a normal XCI pattern. The same variant in the heterozygous state has been previously reported in female patients with OFD type 1 in two cohort studies; however, the maternal phenotype was not addressed in these studies [19,20]. In another study, a family with OFD type 1 was reported, where two female siblings and their mother shared the variant, but only the siblings exhibited severe symptoms [21]. The authors performed an investigation of XCI, which revealed that the mother, who had a moderate phenotype of OFD type 1, displayed a skewed XCI pattern, while her daughter, who had a more severe phenotype, exhibited a random XCI pattern. Additionally, a study conducted in China, published in Chinese, reported female patients with the same variant [22]. However, it was unclear whether normal XCI was observed in the patients or healthy carriers due to our inability to translate the article. In our case, the mother did not exhibit any phenotypic features or symptoms related to OFD type 1 while patient #1 had classical clinical features of OFD type 1. In other studies with XCI with different variants in the *OFD1* gene, and also in patient #1, the mother presented a mild phenotype and a skewed X-inactivation pattern, whereas the daughter displayed a severe phenotype with random X-inactivation [23]. On the contrary, in a different familial case, the mildly symptomatic mother displayed a random inactivation while her daughter with the full OFD type 1 phenotype showed skewed XCI [24].

However, it is also important to consider that the *OFD1* gene has been shown to escape X-inactivation, leading to biallelic expression from both X chromosomes in female cells [2]. Some researchers have suggested that this phenomenon could be further explored by studying *OFD1* gene expression in different tissues [5].

Patient #2 presented with a complex phenotype linked to the hemizygous likely pathogenic variant p.(Gln892Ter) in the *OFD1* gene. He exhibited features consistent with SGBS2, including obesity, intellectual disability, brachydactyly, and PCD—a combination previously described in only one case, an 11-year-old boy, published in 2006 [13]. It is important to note that subsequent studies have not confirmed an association between SGBS2 and variants in the *OFD1* gene, with some researchers suggesting that this link might be a mistake [5,14]. Based on the detailed clinical description of our proband and the absence of pathogenic variants in other relevant genes, we consider our patient to align with the previously described case. However, the accuracy of the term “SGBS2” for our patient and the earlier case remains unclear, as certain features deviate from the classical presentation of SGBS1 associated with variants in the *GPC3* gene [25]. Furthermore, considering that male patients with hemizygous LoF variants in *OFD1* are considered to be embryonically lethal, it is challenging to classify our patient as having OFD type 1 with PCD [5].

The spectrum of *OFD1*-associated disorders encompasses a wide range of syndromes, which can sometimes clinically overlap [5]. Notably, some studies suggest that certain LoF variants located in the last exons of the *OFD1* gene may escape nonsense-mediated decay (NMD), leading to phenotypes such as Joubert syndrome or isolated PCD [7,26]. Although limited research has investigated NMD at the RNA or protein level, evidence exists for variants such as p.Glu933Ter associated with Joubert syndrome with PCD, and p.Lys950Ter linked to Joubert syndrome [26,27]. Both variants were shown to escape NMD. The identified variant, p.(Gln892Ter) in patient #2, may result in the synthesis of a truncated protein, potentially affecting OFD1 protein function differently than what is observed in the cases of PCD or Joubert syndrome. This hypothesis warrants further investigation in future studies.

In the third patient, with a likely pathogenic variant p.(Glu879Ter) in the *OFD1* gene, an isolated form of PCD was identified, without any associated syndromic pathology. Similar observations have been reported in recent studies involving patients with PCD [26,28,29]. In all these cases, the variants were located in the last exons of the gene, suggesting an NMD escape mechanism [30].

## 5. Conclusions

This work contributes to the growing body of literature that recognizes the expanding phenotypic spectrum of *OFD1*-associated conditions, advocating for ongoing exploration into the genetic and clinical implications of *OFD1* variants. Further research is essential to elucidate the molecular mechanisms underlying these syndromes, as understanding the spectrum of *OFD1*-associated disorders may facilitate more focused genetic testing and timely management of associated complications.

## Data Availability

The datasets for this article are not publicly available due to concerns regarding participant/patient anonymity. Requests to access the datasets should be directed to the corresponding author.
